# A key risk indicator approach to central statistical monitoring in multicentre clinical trials: method development in the context of an ongoing large-scale randomized trial

**DOI:** 10.1186/1745-6215-12-S1-A135

**Published:** 2011-12-13

**Authors:** Valdés-Márquez Elsa, Hopewell C Jemma, Landray Martin, Armitage Jane

**Affiliations:** 1 Clinical Trial Service Unit and Epidemiological Studies Unit University of Oxford, Oxford, UK

## Background

Monitoring in randomized trials is recommended as part of International Conference on Harmonisation - Good Clinical Practice standards. On-site monitoring in multicentre trials is common but is costly and can be inefficient. Central statistical monitoring can be used to detect unusual data patterns, identify intentional or unintentional trial misconduct, and to prioritise on-site visits and additional training. Motivated by an ongoing international multicentre clinical trial of over 25,000 randomized participants with electronic data capture, we developed key risk indicator (KRI) methods for central statistical monitoring in multicentre trials.

## Method and results

Statistical monitoring of KRIs such as the rate of serious adverse event reporting, compliance with study treatment visit duration (based on how long the electronic case report form is open for) and blood results may be informative for identifying poor site performance in randomized trials. We have used these KRIs to illustrate an approach to central statistical monitoring.

For examining the rate of serious adverse event reporting, centres were assigned a dichotomous score depending on whether they showed extreme deviation from comparable sites (arbitrarily defined as half the observed median rate across sites). A similar approach was used to identify sites with short visit duration and these methods were also extended to examine repeated measurements of compliance with study treatment.

Blood results and other similar continuous variables were examined for unusual patterns that may suggest fabricated data or training issues. Residuals were calculated from linear regression models allowing for important covariates. The moments of the distribution (mean, variance, skewness and kurtosis) of the residuals within a centre were compared with those across sites. Subsequently, centres were assigned a dichotomous score depending on whether the observed data showed relevant (clinically or otherwise) deviation from comparable sites.

Varying combinations of KRIs may be informative for different questions of interest. For example, KRIs that are useful for identifying potential data fabrication may not be relevant for assessing unintentional protocol violations. Consequently, we used flexible weighting methods to formulate summary scores involving different KRIs of interest.

## Conclusions

KRI methods provide a flexible approach to central statistical monitoring in multicentre clinical trials. However, a KRI approach needs to be tailored to each study, and the KRIs selected using knowledge of what is important to individual clinical trials.

